# Prognostic value of exercise capacity in incident diabetes: a country with high prevalence of diabetes

**DOI:** 10.1186/s12902-022-01174-5

**Published:** 2022-11-30

**Authors:** Abdelrahman A. Jamiel, Husam I. Ardah, Amjad M. Ahmed, Mouaz H. Al-Mallah

**Affiliations:** 1 King Abdulaziz Cardiac Center - Adult Cardiology, King Abdulaziz Medical City for National Guard, 1413 P.O. Box 22490, 11426 Riyadh, Kingdom of Saudi Arabia; 2 King Abdullah International Medical Research Centre, Riyadh, Saudi Arabia; 3 King Saud bin Abdulaziz University for Health Specialties, Riyadh, Saudi Arabia; 4 Department of Biostatistics and bioinformatics, King Abdullah International Medical Research Centre, Riyadh, Saudi Arabia; 5 Ministry of National Guard-Health Affairs, Riyadh, Saudi Arabia; 6 Houston Methodist DeBakey Heart & Vascular Center, Houston, TX USA; 7 Houston Methodist Academic Institute, Houston, TX USA

**Keywords:** Incident Diabetes, Exercise Capacity, Metabolic equivalent of tasks (METs), Net reclassification index, Predictive modeling

## Abstract

**Background:**

Diabetes Mellitus (DM) is a fast-growing health problem that imposes an enormous economic burden. Several studies demonstrated the association between physical inactivity and predicting the incidence of diabetes. However, these prediction models have limited validation locally. Therefore, we aim to explore the predictive value of exercise capacity in the incidence of diabetes within a high diabetes prevalence population.

**Methodology:**

A retrospective cohort study including consecutive patients free of diabetes who underwent clinically indicated treadmill stress testing. Diabetic patients at baseline or patients younger than 18 years of age were excluded. Incident diabetes was defined as an established clinical diagnosis post-exercise testing date. The predictive value of exercise capacity was examined using Harrell’s c-index, net reclassification index (NRI), and integrated discrimination index (IDI).

**Results:**

A total of 8,722 participants (mean age 46 ± 12 years, 66.3% were men) were free of diabetes at baseline. Over a median follow-up period of 5.24 (2.17–8.78) years, there were 2,280 (≈ 26%) new cases of diabetes. In a multivariate model adjusted for conventional risk factors, we found a 12% reduction in the risk of incident diabetes for each METs achieved (HR, 0.9; 95% CI, 0.88–0.92; P < 0.001). Using Cox regression, exercise capacity improved the prediction ability beyond the conventional risk factors (AUC = 0.62 to 0.66 and c-index = 0.62 to 0.68).

**Conclusion:**

Exercise capacity improved the overall predictability of diabetes. Patients with reduced exercise capacity are at high risk for developing incidence diabetes. Improvement of both physical activity and functional capacity represents a preventive measure for the general population.

**Supplementary Information:**

The online version contains supplementary material available at 10.1186/s12902-022-01174-5.

## Background

Diabetes is a major socio-economic health problem, with prevalence expanding globally over the past decades[1] making diabetes prevention a crucial health priority[2, 3]. Strong evidence engaging higher levels of exercise capacity and lifestyle interventions has shown a protective impact on subsequent diabetes[3, 4]. Physical activity influences cardiorespiratory fitness (CRF), which reflects the well-being of the cardiovascular system and muscular strength[5]. The significance of this evidence has led several organizations, including the American Heart Association, the American Diabetes Association, and the US. Department of Health and Human Services to include exercise capacity in their recommendations and guidelines[6, 7]. However, it remains controversial how other diabetes-related risk factors, especially metabolic syndrome elements, might alter the association between fitness and incident diabetes.

The actual effect of exercise capacity on diabetes has not been fully explored as most previous data exemplify nations with low to intermediate disease prevalence[8, 9]. These data reflect societies where the culture of exercise is dominant; however, in the Middle East and North Africa (MENA), a rise in prevalence is likely to occur considering low activity levels among citizens.

We are aiming to extend the exploration of the predictive value of exercise capacity in incidence diabetes by (1) examining the relationship between exercise capacity and incident diabetes among patients free of diabetes at baseline and (2) examining whether the relationship between CRF and incident diabetes differed across demographic characteristics and other traditional diabetes-related risk factors. Furthermore, we assess the relationship between Duke Treadmill Score, exercise capacity, and incidence of diabetes.

## Methods

### Study design and population

A retrospective cohort study included patients who underwent clinically indicated exercise treadmill stress testing at King Abdulaziz cardiac center between April 2001 and December 2016. King Abdulaziz Cardiac Center is a part of a large well-connected health care system providing medical service through primary health care up to the tertiary level which makes patients tracking accessible [10]. The patient’s demographics, medical history, and medications used before the stress test were obtained by reviewing the electronic medical record and database search using the ICD-9 coding system. The exercise stress testing system (MUSE) was used to extract exercise test results. Diabetes at the baseline, young patients (< 18 years), records with incomplete stress testing information, and non-Bruce protocol exercise stress testing were excluded from the study analysis. The study was conducted in full accordance with the protocol and the current revision of the declaration of Helsinki, the Good Clinical Practice. The study was a part of the Saudi CArdioRespiratory Fitness (SCARF) project (Study protocol: RC16/103/R - approved by King Abdullah International Medical Research Center (KAIMRC)).

### Exercise treadmill stress testing

Patients underwent symptom-limited maximal treadmill stress testing, which followed the standard Bruce protocol. The test day was pointed as the individual study baseline. Individual results of the initial exercise test were included in the database. Resting both heart rate and blood pressure were measured in the seated position and recorded immediately before each test. Supervised clinicians were following American Heart Association/American College of Cardiology (AHA/ACC) guidelines for terminating the test if the patient had Exercise limiting symptoms: chest pain, shortness of breath, significant arrhythmias, abnormal hemodynamic responses, diagnostic ST-segment changes, other limiting symptoms independent of the achieved heart rate or if the participant was unwilling or unable to continue. Otherwise, patients could reach their peak attainable workload independent of the heart rate achieved. Target heart rate was calculated as 85% of the age-predicted maximal heart rate; the patient’s age was subtracted from a constant value of 220. Metabolic equivalents (METs) were adopted to represent cardiorespiratory fitness status based on the workload derived from the maximal speed and grade achieved during the total treadmill time. METs results were categorized into four groups: <6, 6–9, 10–11, and ≥ 12 METs.

### Study definitions for risk factors

The history of hypertension was defined as a prior diagnosis of hypertension or the use of antihypertensive medications at the time of stress testing. Dyslipidemia was defined as the prior diagnosis of any significant lipid abnormality in the medical records or lipid-lowering medication use. On a prior angiogram, patients with obstructive coronary artery disease (CAD), prior myocardial infarction, coronary angioplasty, or coronary artery bypass surgery are considered known coronary artery disease. Prior congestive heart failure was defined as a prior clinical diagnosis of systolic or diastolic heart failure.

### Study outcome: Incident Diabetes

Incident diabetes was determined among patients without diabetes at baseline and defined as clinical diagnosis of diabetes in the medical records or clinical problem list, use of anti-hyperglycemic medications including insulin, or had lab results suggestive of diabetes post-exercise testing date without further details on the type of diabetes. Time-to-incident diabetes was based on the time between treadmill testing and the date of the first encounter with a new diabetes diagnosis.

### Statistical analysis plan

Study participants were divided into four groups based on their METs (< 6, 6–9, 10–11, and ≥ 12). Categorical variables were presented in frequencies and percentages. Continuous variables were presented based on the normality of distribution as mean ± standard deviation or median and interquartile ranges, as appropriate. The four groups were compared using Chi-square or Fisher exact test for categorical variables. Analysis of Variance (ANOVA) and Kruskal Wallis test was used for continuous variables compersion, as appropriate. Cumulative incidence was presented at 5-, 10-, and 15-yr intervals via a bar graph.

Kaplan-Meier cumulative incident diabetes was computed for different exercise capacity groups, and they were compared using the log-rank test. Cox regression was used to compute hazard ratios (HR) and 95% confidence intervals (CI). A forward selection technique was used to demonstrate independent predictors of incident diabetes. In each forward step, we added a related set of variables to improve our model. Gender, age, and heart rate were included in the baseline model (model 1). Subsequently, cardiovascular risk factors (model 2), Medications used (model3), and exercise stress testing findings (model 4). We computed Harrell’s concordance index (C-index) area under the Curve (AUC) and Akaike information criterion (AIC) to compare the models using this methodology developed for the incidence of diabetes. The selection of variables for entry consideration was based on clinical judgment, results of previous publications, and the expertise of the investigators. We also plotted a restricted cubic spline model to show the shape of the continuous relationship between METs and incident diabetes after adjustment for covariates. Finally, we examined the association between METs and incident diabetes in the subset of participants with a BMI measurement (N = 6,539) (Supplementary Table 1). All Statistical analyses were conducted using SAS 9.4 (SAS Institute Inc., Cary, NC, USA). Statistical significance was defined as *P* ≤ 0.05.

## Results

Our study included 8,722 participants without diabetes at baseline. The mean age of study participants was 46 ± 12 years, and 66.5% were men; 33.9% were hypertensive; 28.3% had hyperlipidemia; 5.4% were known to have prior coronary artery disease (CAD), and only a few of them had heart failure (Table 1).

**Table 1:** Baseline characteristics of the study cohort

	Total	METs < 6	METs 6–9	METs 10–11	METs ≥ 12	***p***-value
	8722	589 (6.7%)	3077 (35.3%)	2930 (33.6%)	2126 (24.4%)	
Age (year)	45.9 ± 12.06	50.9 ± 12.37	48.4 ± 11.87	45.5 ± 11.81	41.3 ± 10.96	< 0.0001
Gender (Male)	5798 (66.5%)	254 (43.1%)	1410 (45.8%)	2157 (73.6%)	1977 (93.0%)	< 0.0001
Height (cm)	162.2 ± 19.51	157.8 ± 18.52	159.4 ± 17.97	164.2 ± 17.85	165.0 ± 23.49	< 0.0001
Weight (kg)	79.7 ± 16.55	81.7 ± 18.08	81.3 ± 17.51	79.5 ± 16.52	77.2 ± 14.11	< 0.0001
BMI (kg/m^2^)	29.4 ± 6.15	32.3 ± 7.22	31.2 ± 6.58	28.5 ± 5.23	26.9 ± 5.10	< 0.0001
**Cardiovascular risk factors**						
Hypertension	2955 (33.9%)	258 (43.8%)	1154 (37.5%)	987 (33.7%)	556 (26.2%)	< 0.0001
Hyperlipidaemia	2465 (28.3%)	172 (29.2%)	880 (28.6%)	884 (30.2%)	529 (24.9%)	0.0005
Known CAD	474 (5.4%)	27 (4.6%)	157 (5.1%)	184 (6.3%)	106 (5.0%)	0.0938
Known CHF	66 (0.8%)	4 (0.7%)	30 (1.0%)	23 (0.8%)	9 (0.4%)	0.1593
Prior CABG	131 (1.5%)	10 (1.7%)	57 (1.9%)	43 (1.5%)	21 (1.0%)	0.0885
Prior MI	454 (5.2%)	30 (5.1%)	151 (4.9%)	172 (5.9%)	101 (4.8%)	0.2524
Prior PCI	306 (3.5%)	16 (2.7%)	92 (3.0%)	123 (4.2%)	75 (3.5%)	0.0538
Smoking	277 (3.2%)	34 (5.8%)	88 (2.9%)	107 (3.7%)	48 (2.3%)	< 0.0001
Lung disease	348 (4.0%)	37 (6.3%)	145 (4.7%)	124 (4.2%)	42 (2.0%)	< 0.0001
Lab results*						
Cholesterol	4.6 (3.87, 5.30)	4.4 (3.80, 5.20)	4.5 (3.84, 5.28)	4.5 (3.87, 5.33)	4.6 (3.95, 5.35)	0.0142
Triglyceride	1.4 (0.99, 1.86)	1.4 (1.04, 1.94)	1.3 (0.98, 1.82)	1.4 (0.98, 1.89)	1.4 (1.02, 1.91)	0.0128
HDL	1.0 (0.88, 1.22)	1.0 (0.87, 1.22)	1.1 (0.90, 1.25)	1.0 (0.87, 1.22)	1.0 (0.88, 1.18)	< 0.0001
LDL	2.8 (2.22, 3.49)	2.7 (2.10, 3.32)	2.8 (2.19, 3.43)	2.8 (2.22, 3.51)	2.9 (2.29, 3.62)	< 0.0001
Haemoglobin	143 (129, 154)	133.0 (121, 144)	136.0 (123, 148)	146.0 (132, 156)	152.0 (142, 160)	< 0.0001
Haematocrit	0.4 (0.39, 0.46)	0.4 (0.37, 0.43)	0.4 (0.37, 0.44)	0.4 (0.40, 0.46)	0.4 (0.42, 0.47)	< 0.0001
Calcium	2.3 (2.25, 2.40)	2.3 (2.20, 2.35)	2.3 (2.23, 2.39)	2.3 (2.25, 2.39)	2.4 (2.28, 2.42)	< 0.0001
BUN	4.6 (3.80, 5.80)	4.5 (3.60, 6.00)	4.5 (3.60, 5.80)	4.7 (3.80, 5.80)	4.8 (4.00, 5.80)	< 0.0001
Creatinine	75.0 (64.00, 89.00)	69.5 (60.00, 85.00)	70.0 (60.00, 84.00)	75.0 (65.00, 88.00)	81.0 (72.00, 93.00)	< 0.0001
eGFR	94.7 (80.39, 109.42)	92.8 (77.29, 106.46)	93.7 (78.85, 107.83)	95.8 (81.84, 110.79)	94.3 (81.44, 110.09)	0.0001
hsCRP	2.5 (0.93, 5.00)	6.0 (2.78, 11.00)	3.5 (1.24, 6.16)	2.7 (0.85, 5.00)	1.4 (0.78, 2.38)	0.0001
Vitamin-D	32.9 (23.00, 48.00)	29.8 (21.00, 47.00)	33.0 (22.20, 49.00)	33.3 (23.15, 49.40)	32.6 (23.90, 46.00)	0.0001
**Medications**						
Beta-blockers	710 (8.1%)	61 (10.4%)	268 (8.7%)	247 (8.4%)	134 (6.3%)	0.0017
Calcium channel blockers	503 (5.8%)	54 (9.2%)	202 (6.6%)	159 (5.4%)	88 (4.1%)	< 0.0001
ACEI	512 (5.9%)	40 (6.8%)	177 (5.8%)	191 (6.5%)	104 (4.9%)	0.0753
ARB	988 (11.3%)	86 (14.6%)	386 (12.5%)	341 (11.6%)	175 (8.2%)	< 0.0001
ACEARB	1288 (14.8%)	107 (18.2%)	489 (15.9%)	452 (15.4%)	240 (11.3%)	< 0.0001
Aspirin	875 (10.0%)	60 (10.2%)	309 (10.0%)	328 (11.2%)	178 (8.4%)	0.0123
Digoxin	48 (0.6%)	7 (1.2%)	20 (0.6%)	12 (0.4%)	9 (0.4%)	0.0847
PPI	744 (8.5%)	59 (10.0%)	271 (8.8%)	284 (9.7%)	130 (6.1%)	< 0.0001
Plavix	2057 (23.6%)	149 (25.3%)	718 (23.3%)	717 (24.5%)	473 (22.2%)	0.2169
Statin	1388 (15.9%)	86 (14.6%)	483 (15.7%)	506 (17.3%)	313 (14.7%)	0.0674
Diuretic	357 (4.1%)	41 (7.0%)	154 (5.0%)	110 (3.8%)	52 (2.4%)	< 0.0001
**Stress test results**						
Rest HR (bpm)	81.9 ± 16.12	86.9 ± 18.45	84.8 ± 17.00	81.1 ± 15.05	77.5 ± 14.21	< 0.0001
Peak HR (bpm)	155.5 ± 22.13	130.9 ± 25.28	149.8 ± 21.76	158.5 ± 18.79	166.7 ± 17.53	< 0.0001
Rest SBP (mmHg)	129.8 ± 16.94	133.0 ± 19.06	130.7 ± 18.34	129.1 ± 16.04	128.4 ± 14.98	< 0.0001
Peak SBP (mmHg)	160.8 ± 25.92	152.7 ± 32.48	160.7 ± 28.64	162.4 ± 24.55	160.7 ± 21.29	< 0.0001
Rest DBP (mmHg)	79.2 ± 9.88	79.5 ± 10.07	78.6 ± 10.38	79.6 ± 9.45	79.5 ± 9.62	0.0034
Peak DBP (mmHg)	82.8 ± 10.63	82.3 ± 10.89	83.4 ± 10.77	83.4 ± 10.76	81.2 ± 10.06	< 0.0001
Peak METs	10.1 ± 2.80	4.7 ± 0.95	8.0 ± 1.04	10.7 ± 0.62	13.6 ± 1.48	< 0.0001
Chronotropic Incompetence	2275 ( 26.1% )	391 ( 66.4% )	1013 ( 32.9% )	591 ( 20.2% )	280 ( 13.2% )	< 0.0001
Duke Treadmill Score	5.5 ± 6.12	1.8 ± 5.79	4.0 ± 5.43	6.2 ± 5.73	7.9 ± 6.56	< 0.0001
Low	6040 (69.3%)	114 (19.4%)	2064 (67.1%)	2221 (75.8%)	1641 (77.2%)	< 0.0001
Moderate	2447 (28.1%)	454 (77.1%)	925 (30.1%)	597 (20.4%)	471 (22.2%)	
High	235 (2.7%)	21 (3.6%)	88 (2.9% )	112 (3.8% )	14 (0.7%)	

**CAD**: Coronary artery disease, **CHF**: Congestive heart failure, **CABG**: Coronary artery bypass grafting, **MI**: Myocardial infarction, **PCI**: Percutaneous coronary intervention, **HDL**: High density lipoprotein, **LDL**: Low density lipoprotein, **BUN**: Blood urea nitrogen, **eGFR**: estimated Glomerular filtration rate, **ACEI**: Angiotensin converting enzyme inhibitors, **ARB**: Angiotensin Receptor blocker, **PPI**: Proton pump inhibitors, **HR**: Heart rate, **SBP**: Systolic blood pressure, **DBP**: Diastolic blood pressure, **METs**: Metabolic equivalent of tasks

Participants with the highest exercise capacity (≥ 12METs) were younger (41 ± 3 vs. 51 ± 12 years, *p* < 0.001); more often males (91% versus 43%, *p* < 0.001); had lower mean body mass index (26 ± 9 vs. 32 ± 3 kg/m^2^, *p* < 0.001) and less likely to have hypertension or hyperlipidemia (26% vs. 44% and 25% vs. 29%, respectively, *p* < 0.001) in comparison with the lowest exercise capacity group (METs < 6). No apparent differences between CRF categories regarding prior coronary artery disease (CAD) and heart failure were observed. At peak exercise, the heart rate was higher among the highest achievers (166 ± 2 vs. 131 ± 25 bpm, *p* < 0.001) as well as systolic blood pressure (161 ± 21 vs. 153 ± 33 mmHg, *p* < 0.001). Moreover, the highest METs-achieved was associated with a lower risk of Duke Treadmill risk, while high Duke Treadmill risk was observed in those with lower METs achieved (for low Duke score: 77.2% vs. 19.4%) and (for high Duke score 0.7% vs. 2.7%), *p* < 0.001.

**Fig. 1:**
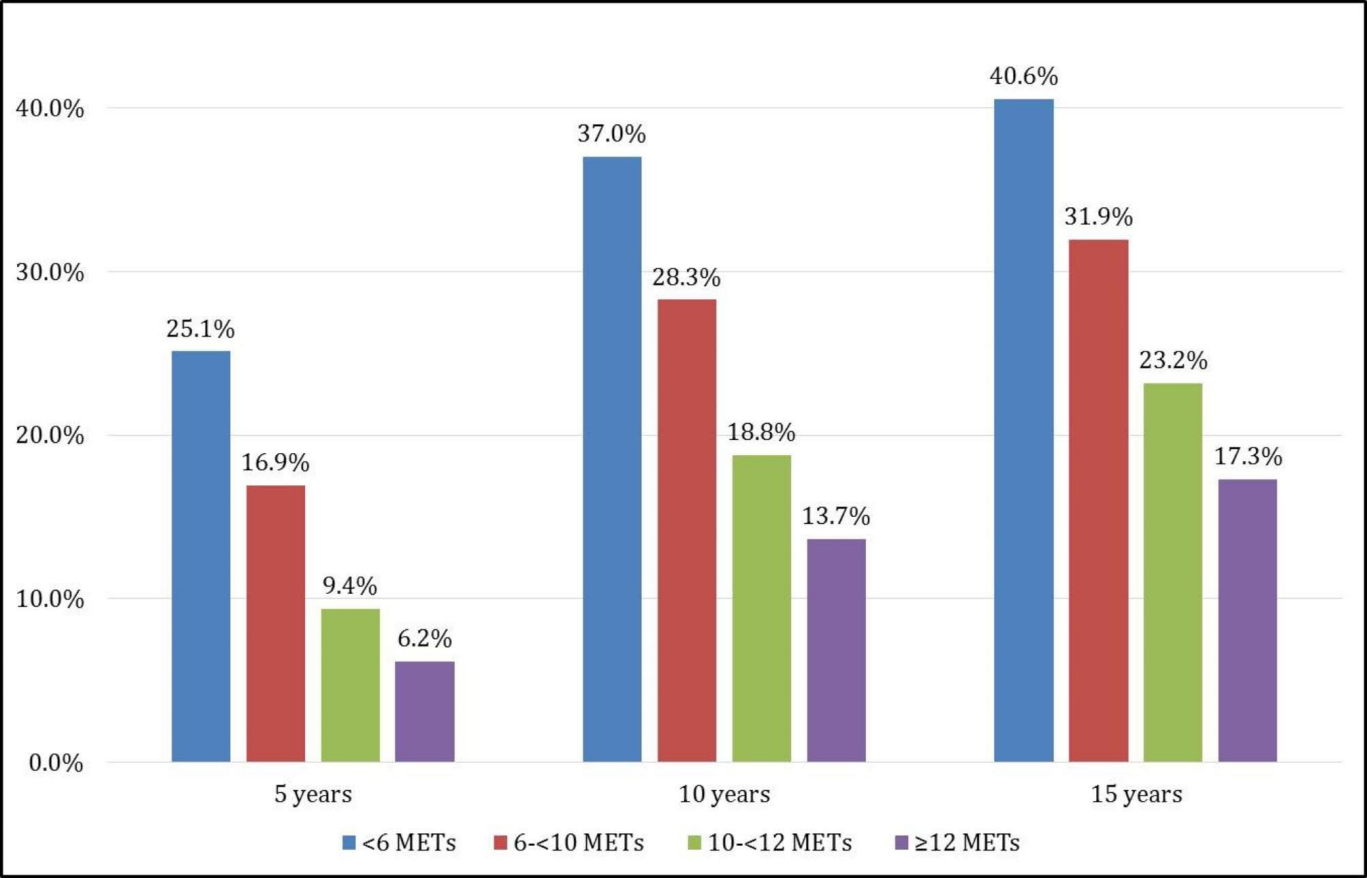
The cumulative rates of incidence diabetes

Over a median follow-up period of 5.24 (IQR: 2.17–8.78) years, there were 2,280 (25.96%) new cases of diabetes. The unadjusted 5-year accumulative incidence rates of diabetes across categories of CRF (< 6, 6 < 9, 10 < 11, and ≥ 12 METs) were 25.1%, 16.9%, 9.4%, and 6.2%, respectively. The cumulative rates of incidence of diabetes by the end of 15 year follow-up period were as follows: 40.6%, 31.9%, 23.2%, and 17.3% for those who achieved < 6, 6 < 9, 10 < 11, and ≥ 12 METs, respectively (Fig. 1).

**Fig. 2:**
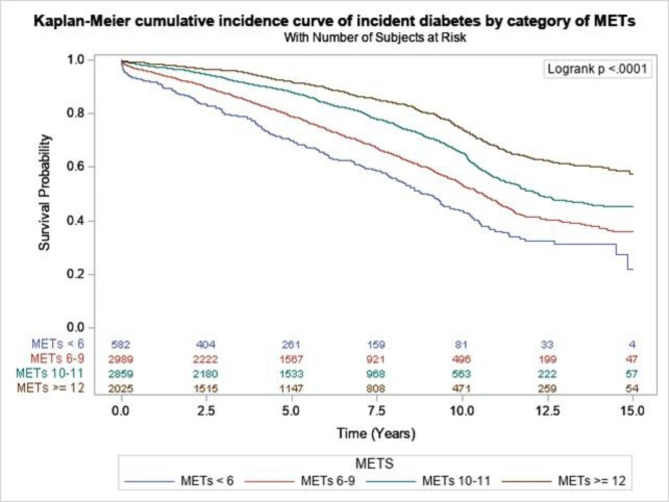
Kaplan-Meier cumulative incidence curve and the trends across categories of METs

Examination of the crude association between categories of METs and risk for incident diabetes using a Kaplan-Meier cumulative incidence curve revealed a significant trend across categories of METs (log-rank < 0.001) (Fig. 2). In a multivariate Cox regression model adjusted for potential confounders, we found a 10% reduction in the risk of incident diabetes with higher METs achieved (HR, 0.90; 95% CI, 0.88–0.92; *p* < 0.001) (model 4B) and risk for incident diabetes reduced per each METs achieved; 13% (6 < 9 METs), 38% (10 < 11), and 52% (≥ 12 METs) compare to lower METs (< 6 METs) *p* < 0.001. Adding the exercise capacity information (METs achieved, CI, and Duke Risk score) improves the overall predictability of the model (model 4 A) over the baseline model (Table 2A). Examining the predictive value of the finding of exercise stress testing (using AIC, C-statistics, IAUC, NRI, and IDI), we found superiority of METs groups in the prediction incidence of diabetes above the other models resulting in a significant reclassification of the study cohort and significant improvement of the area under the curve above the primary model (Table 2B).

**Table 2A:** Adjusted multivariate Cox regression model for exercise stress testing

	Model (1)		Model (2)		Model (3)		Model (4 A)		Model (4B)	
	HR (95% CI)	***p***	HR (95% CI)	***p***	HR (95% CI)	***p***	HR (95% CI)	***p***	HR (95% CI)	***p***
Age (years)	1.032 (1.03–1.04)	**< 0.001**	1.028 (1.02–1.03)	**< 0.001**	1.028 (1.02–1.03)	**< 0.001**	1.022 (1.02–1.03)	**< 0.001**	1.02 (1.02–1.02)	**< 0.001**
Gender (Female vs. Male)	1.131 (1.03–1.24)	**0.009**	1.176 (1.07–1.29)	**0.001**	1.138 (1.04–1.25)	**0.007**	0.892 (0.81–0.99)	**0.027**	0.87 (0.79–0.96)	**0.007**
Resting heart rate (bpm)	1.002 (1.00–1.00)	**0.109**	1.003 (1.00-1.01)	**0.011**	1.004 (1.00-1.01)	**0.003**	1.002 (1.00-1.01)	**0.109**	1.001 (1.00–1.00)	**0.452**
Cardiovascular risk factors										
Hypertension			1.189 (1.08–1.31)	**< 0.001**	1.138 (1.03–1.26)	**0.015**	1.06 (0.95–1.18)	**0.284**	1.059 (0.95–1.18)	**0.293**
Hyperlipidemia			1.196 (1.08–1.32)	**< 0.001**	1.502 (1.36–1.66)	**< 0.001**	1.537 (1.39–1.70)	**< 0.001**	1.539 (1.39–1.71)	**< 0.001**
Smoking			1.202 (0.94–1.54)	**0.150**	1.241 (0.97–1.59)	**0.090**	1.19 (0.93–1.52)	**0.167**	1.178 (0.92–1.51)	**0.193**
Lung disease			1.06 (0.77–1.46)	**0.720**	0.961 (0.69–1.34)	**0.814**	0.94 (0.67–1.31)	**0.716**	0.908 (0.65–1.27)	**0.572**
Known CAD			1.154 (0.98–1.36)	**0.092**	1.149 (0.97–1.37)	**0.117**	1.18 (0.99–1.40)	**0.060**	1.189 (1.00-1.41)	**0.049**
Known CHF			0.588 (0.36–0.97)	**0.039**	0.483 (0.29–0.81)	**0.005**	0.431 (0.26–0.72)	**0.001**	0.429 (0.26–0.71)	**0.001**
Medications										
Statins					0.332 (0.28–0.40)	**< 0.001**	0.338 (0.28–0.41)	**< 0.001**	0.338 (0.28–0.41)	**< 0.001**
PPI					1.632 (1.32–2.01)	**< 0.001**	1.563 (1.27–1.93)	**< 0.001**	1.568 (1.27–1.94)	**< 0.001**
CCB					0.994 (0.81–1.21)	**0.955**	1.005 (0.82–1.23)	**0.964**	0.999 (0.82–1.22)	**0.993**
BB					1.697 (1.38–2.09)	**< 0.001**	1.615 (1.31–1.99)	**< 0.001**	1.588 (1.29–1.96)	**< 0.001**
ACE/ARB					0.996 (0.86–1.15)	**0.951**	0.979 (0.85–1.13)	**0.771**	0.988 (0.86–1.14)	**0.866**
Diuretic					1.187 (0.95–1.48)	**0.124**	1.182 (0.95–1.47)	**0.138**	1.164 (0.93–1.45)	**0.178**
METs (Categories)										
METs 6–9							0.871 (0.75–1.01)	**0.073**		
METs 10–11							0.619 (0.52–0.73)	**< 0.001**		
METs ≥ 12							0.482 (0.39–0.59)	**< 0.001**		
METs (Continuous)									0.90 (0.88–0.92)	**< 0.001**
Chronotropic incompetence							1.119 (1.01–1.24)	**0.030**	1.056 (0.95–1.17)	**0.303**
Duke Risk Score										
High Risk							1.293 (1.00-1.67)	**0.048**	1.257 (0.97–1.62)	**0.078**
Moderate Risk							1.26 (1.14–1.39)	**< 0.001**	1.21 (1.10–1.33)	**< 0.001**

** h**: Hazard ratio, **CI**: confidence interval, **CAD**: Coronary artery disease, **CHF**: Congestive heart failure, **PPI**: Proton pump inhibitors, **CCB**: Calcium channel blocker, **BB**: beta-blockers, **ACE/ARB**: Angiotensin-converting enzyme/Angiotensin receptor blockers, **METs**: Metabolic equivalent of tasks,

**Table 2B:** Predictive value of the models finding for exercise stress testing

	Model (1)	Model (2)	Model (3)	Model (4 A)	Model (4B)
AIC	36619.287	36573.79	36408.081	36253.129	36227.135
C-Statistics	0.6221	0.6289	0.6581	0.6817	0.6839
IAUC	0.6256	0.6352	0.6581	0.6911	0.6948
NRI	0.3757	0.2451	0.3562	0.1595	0.129
IDI	0.0123	0.0019	0.0065	0.0026	0.0026

**Model [1]:** Age, Gender, Resting heart rate.

**Model [2]:** Model [1] + Cardiovascular risk factors.

**Model (3):** Model (2) + Medications used.

**Model (4A):** Model (3) + Finding of exercise stress testing (METs in categories)

**Model (4B):** Model (3) + Finding of exercise stress testing (METs as continuous)

**AIC**: Akaike information criterion, **C-statistics**: concordance statistic, **IAUC**: Incremental Area Under the Curve, **NRI**: Net reclassification improvement, **IDI**: Integrated Discrimination Index

**Fig. 3:**
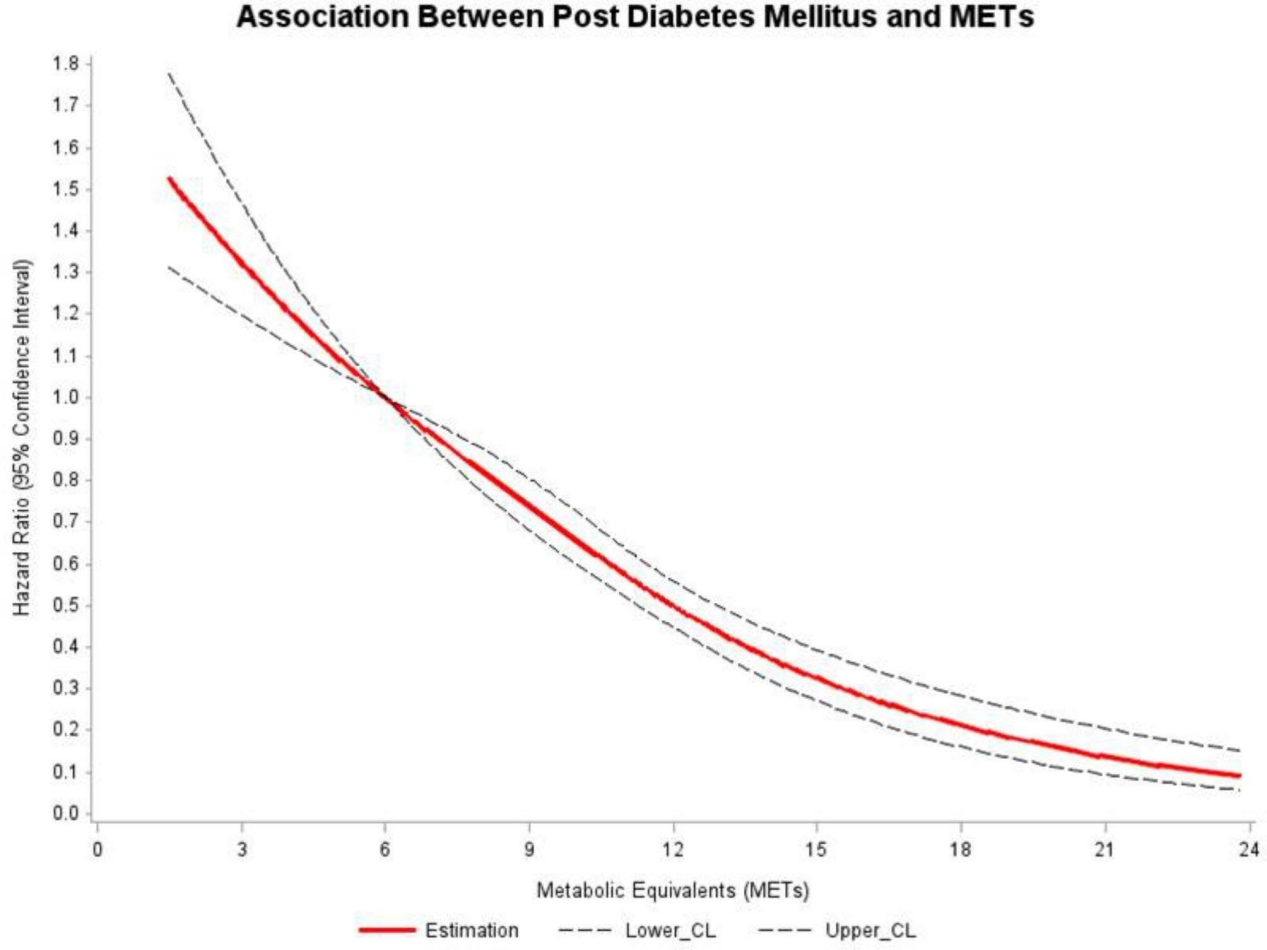
The relationship of incidence diabetes risk observed for every improvement of exercise capacity

A non-linear inverse relationship was observed between baseline exercise capacity and risk of incident diabetes after adjusting for known confounders. A gradual decline in the risk of development of diabetes was observed for every improvement of exercise capacity (METs increase) above 6 METs (Fig. 3).

## Discussion

This study investigated the relationship between exercise capacity and incident diabetes. To the best of our knowledge, it is the first large and demographically diverse cohort to examine the association between CRF estimated by METs using a standard test (Bruce protocol) by exercise treadmill testing and the risk of incident diabetes in the MENA region. It has shown that higher fitness was inversely associated with incident diabetes, such that every increment of fitness by 1 METs was associated with a 12% lower risk of incident diabetes. Another important finding in our study was the inverse linear relationship between CRF and diabetes, which notably did not show evidence of plateauing or reversal at higher levels of CRF regardless of age, sex, or other diabetes risk factors.

The mechanism that links the relationship between fitness and incident diabetes is a subject of much discussion. Individuals with low cardiorespiratory fitness have high insulin resistance[11], and low levels of CRF have fewer glucose transporters[12] compared with those more fit. It is thought to be mediated by positive changes in the human body tissue profile, reduced adiposity[13], and an immediate increase in insulin sensitivity and glucose disposal[14]. However, several studies have shown that impaired oxidative respiration due to mitochondrial dysfunction[15] underlies the pathogenesis of diabetes reflecting a native genetic state independent of physical activity[16].

Scattered small cross-sectional studies have shown that exercise capacity is inversely associated with impaired glycemic control[17], the metabolic syndrome[18], diabetes[19] and positively associated with glucose disposal rate and insulin sensitivity[20]. Unfortunately, these studies have been small and confined to limited demographic settings (single-sex, racial group, or age-group). Others had reported the relationship between CRF level and the incident diabetes in prospective[21–23] or measuring CFR differently[24].

Low CFR in diabetic patients is an independent risk factor for mortality, and it was associated with 2.1 odds of mortality compared with high CFR diabetic patients[25]. Long-standing diabetes leads to consequences of peripheral neuropathy and vasculopathy with the reduction in the activity of daily living, poor physical function, and disability[26–28]. Additionally, concomitant diabetes risk factors expedite the complications’ progression and highlight the need for urgent intervention on several prevention levels[29]. Implementing the WHO recommendations for reducing physical inactivity by 10–15% will gradually reduce T2DM in adults[30]. A 150-min of moderate-intensity exercise weekly was associated with a reduction of T2DM by 26%[31].

### Study limitations and strengths

Our study is not without limitations. First, cardiovascular health is independently associated with behaviors that may represent the causal contributing to fitness. Unfortunately, we are unable to assess these behaviors in our study formally. Second, incident diabetes was based on medical records and administrative claims files, which were not collected initially to examine diabetes. As a result, our study did not include study protocol–based direct measurements of hemoglobin A1c, blood glucose, or oral glucose tolerance testing. Because of this, several people with undiagnosed diabetes may have been included in our study population or missed as incident cases, attenuating our results. Third, our study population comprised persons referred for stress testing, which undoubtedly carries a higher burden of cardiovascular disease at baseline than the general population and may have led to referral bias. That limits our study’s generalizability. Fourth, residual confounding is always a concern with observational studies, especially with covariates assessed via the medical record rather than a direct measurement. Finally, our study reports a single center’s experience with its unique practice patterns and mode of operation. Thus, although the cohort studied was diverse, it may not represent the entire adult population of Saudi Arabia.

On the other hand, our study has some strengths. The standard stress testing, the Bruce protocol treadmill test, characterized fitness rather than self-reporting physical activity. This way is readily interpreted in clinical settings. Furthermore, our study sample was a large and diverse sample, which adequately powered our study.

## Conclusion

In conclusion, our results showed a strong inverse relationship between cardiorespiratory fitness and the development of type 2 diabetes. This relationship was independent of other traditional risk factors for diabetes. This study provides further supporting evidence of the benefits of higher overall fitness. Maintaining a high cardiorespiratory fitness level may contribute to the prevention of type 2 diabetes; therefore, we hope our findings will encourage health professionals to advise the general public and diabetics to lead an active lifestyle and improve their fitness levels. Prospective future studies are required to evaluate these findings further.

## Electronic supplementary material

Below is the link to the electronic supplementary material.

Supplementary Material 1

## Data Availability

The datasets generated are not for publicly sharing according to the policy declared by King Abdullah International Medical Research Center in Riyadh-Saudi Arabia and a data sharing agreement has to be signed for that with King Abdullah International Medical Research Center through the corresponding author on reasonable request.

## Abbreviations

DM: Diabetes mellitus
CRF: Cardiorespiratory fitness
MENA: Middle East and North Africa
ICD: International Classification of Diseases
SCARF project: Saudi CArdioRespiratory Fitness project
KAIMRC: King Abdulla International Medical Research Center
AHA/ACC: American Heart Association/American College of Cardiology
METs: Metabolic equivalents of tasks
CAD: Coronary artery disease
ANOVA: Analysis of Variance
HR: Hazard ratios
CI: confidence intervals
AIC: Akaike information criterion
AUC: Area under the Curve
C-index: Concordance index
IDI: Integrated discrimination index
AIC: Akaike information criterion
C-statistics: concordance statistic
IAUC: Incremental Area Under the Curve
NRI: Net reclassification improvement
IDI: Integrated Discrimination Index
