# Supplementary material for: Prognostic value of exercise capacity in incident diabetes: a country with high prevalence of diabetes
Source: BMC Endocr Disord. 2022 Nov 30;22:297. doi: 10.1186/s12902-022-01174-5 (PMC9710054; doi:10.1186/s12902-022-01174-5)
Supplement: Supplementary file 1 — Supplementary Material 1 [file 12902_2022_1174_MOESM1_ESM.docx]

| **Supplementary Table (1)** The association between METs and incident diabetes in the subset of participants with a BMI measurement | | | | | | | | | | |
| --- | --- | --- | --- | --- | --- | --- | --- | --- | --- | --- |
|  | **Model (1)** | | **Model (2)** | | **Model (3)** | | **Model (4A)** | | **Model (4B)** | |
| AIC | 29837.87 | | 29812.71 | | 29749.16 | | 29663.62 | | 29647.19 | |
| C-Statistics | 0.6253 | | 0.6266 | | 0.6412 | | 0.6626 | | 0.6639 | |
| IAUC | 0.6161 | | 0.6199 | | 0.6336 | | 0.666 | | 0.6689 | |
| NRI | 0.5109 | | 0.2554 | | 0.3419 | | 0.0657 | | 0.0457 | |
| IDI | 0.0369 | | 0.0023 | | 0.004 | | 0.0011 | | 0.0009 | |
|  | HR (95% CI) | ***p*** | HR (95% CI) | ***p*** | HR (95% CI) | ***p*** | HR (95% CI) | ***p*** | HR (95% CI) | ***p*** |
| Age (years) | 1.030 (1.03-1.03) | **<.001** | 1.027 (1.02-1.03) | **<.001** | 1.027 (1.02-1.03) | **<.001** | 1.022 (1.02-1.03) | **<.001** | 1.021 (1.02-1.03) | **<0.001** |
| Gender (Female vs Male) | 0.947 (0.86-1.05) | **0.291** | 0.983 (0.89-1.09) | **0.738** | 0.958 (0.86-1.06) | **0.421** | 0.802 (0.72-0.90) | **<.001** | 0.783 (0.70-0.88) | **<0.001** |
| BMI | 1.029 (1.02-1.04) | **<.001** | 1.029 (1.02-1.04) | **<.001** | 1.030 (1.02-1.04) | **<.001** | 1.022 (1.01-1.03) | **<.001** | 1.021 (1.01-1.03) | **<0.001** |
| Resting heart rate (bpm) | 1.004 (1.00-1.01) | **0.008** | 1.005 (1.00-1.01) | **0.001** | 1.005 (1.00-1.01) | **0.001** | 1.004 (1.00-1.01) | **0.012** | 1.003 (1.00-1.01) | **0.059** |
| Cardiovascular risk factors |  |  |  |  |  |  |  |  |  |  |
| Hypertension |  |  | 1.125 (1.01-1.25) | **0.027** | 1.081 (0.97-1.21) | **0.173** | 1.021 (0.91-1.15) | **0.726** | 1.021 (0.91-1.15) | **0.72** |
| Hyperlipidemia |  |  | 1.229 (1.10-1.37) | **0** | 1.401 (1.25-1.56) | **<.001** | 1.426 (1.28-1.59) | **<.001** | 1.428 (1.28-1.59) | **<0.001** |
| Smoking |  |  | 1.135 (0.87-1.48) | **0.354** | 1.193 (0.91-1.56) | **0.202** | 1.155 (0.88-1.51) | **0.293** | 1.14 (0.87-1.49) | **0.335** |
| Lung disease |  |  | 0.960 (0.69-1.34) | **0.81** | 0.900 (0.64-1.27) | **0.552** | 0.891 (0.63-1.26) | **0.513** | 0.869 (0.61-1.23) | **0.428** |
| Known CAD |  |  | 1.054 (0.88-1.26) | **0.56** | 1.113 (0.92-1.34) | **0.259** | 1.128 (0.94-1.36) | **0.201** | 1.143 (0.95-1.37) | **0.157** |
| Known CHF |  |  | 0.550 (0.33-0.93) | **0.025** | 0.475 (0.28-0.81) | **0.006** | 0.436 (0.26-0.74) | **0.002** | 0.438 (0.26-0.74) | **0.002** |
| Medications |  |  |  |  |  |  |  |  |  |  |
| Statins |  |  |  |  | 0.437 (0.35-0.54) | **<.001** | 0.442 (0.36-0.55) | **<.001** | 0.442 (0.36-0.55) | **<0.001** |
| PPI |  |  |  |  | 1.450 (1.16-1.80) | **0.001** | 1.409 (1.13-1.76) | **0.002** | 1.407 (1.13-1.75) | **0.002** |
| CCB |  |  |  |  | 1.032 (0.82-1.30) | **0.787** | 1.053 (0.84-1.33) | **0.659** | 1.048 (0.83-1.32) | **0.691** |
| BB |  |  |  |  | 1.430 (1.14-1.80) | **0.002** | 1.357 (1.08-1.71) | **0.009** | 1.333 (1.06-1.68) | **0.014** |
| ACE/ARB |  |  |  |  | 0.984 (0.84-1.15) | **0.835** | 0.980 (0.84-1.14) | **0.797** | 0.985 (0.84-1.15) | **0.843** |
| Diuretic |  |  |  |  | 1.199 (0.95-1.51) | **0.123** | 1.194 (0.95-1.51) | **0.133** | 1.177 (0.93-1.48) | **0.169** |
| METs (Categories) |  |  |  |  |  |  |  |  |  |  |
| METs 6-9 |  |  |  |  |  |  | 0.658 (0.54-0.79) | **<.001** |  |  |
| METs 10-11 |  |  |  |  |  |  | 0.890 (0.75-1.05) | **0.17** |  |  |
| METs ≥ 12 |  |  |  |  |  |  | 0.570 (0.46-0.71) | **<.001** |  |  |
| METs (Continuous) |  |  |  |  |  |  |  |  | 0.917 (0.90-0.94) | **<0.001** |
| Chronotropic incompetence |  |  |  |  |  |  | 1.131 (1.01-1.26) | **0.03** | 1.078 (0.96-1.21) | **0.192** |
| Duke Risk Score |  |  |  |  |  |  |  |  |  |  |
| High Risk |  |  |  |  |  |  | 1.299 (0.99-1.70) | **0.058** | 1.267 (0.97-1.66) | **0.086** |
| Moderate Risk |  |  |  |  |  |  | 1.234 (1.11-1.37) | **<.001** | 1.199 (1.08-1.33) | **0.001** |
| **HR**: Hazard ratio, **CI**: confidence interval, **BMI**: Body mass index, **CAD**: Coronary artery disease, **CHF**: Congestive heart failure, **PPI**: Proton pump inhibitors, **CCB**: Calcium channel blocker, **BB**: beta-blockers, **ACE/ARB**: Angiotensin-converting enzyme/Angiotensin receptor blockers, **METs**: Metabolic equivalent of tasks, **AIC**: Akaike information criterion, **C-statistics**: concordance statistic, **IAUC**: Incremental Area Under the Curve, **NRI**: Net reclassification improvement, **IDI**: Integrated Discrimination Index | | | | | | | | | | |
